# Circulating *AR* copy number and outcome to enzalutamide in docetaxel-treated metastatic castration-resistant prostate cancer

**DOI:** 10.18632/oncotarget.9341

**Published:** 2016-05-13

**Authors:** Samanta Salvi, Valentina Casadio, Vincenza Conteduca, Cristian Lolli, Giorgia Gurioli, Filippo Martignano, Giuseppe Schepisi, Sara Testoni, Emanuela Scarpi, Dino Amadori, Daniele Calistri, Gerhardt Attard, Ugo De Giorgi

**Affiliations:** ^1^ Biosciences Laboratory, Istituto Scientifico Romagnolo per lo Studio e la Cura dei Tumori (IRST) IRCCS, Meldola, Italy; ^2^ Department of Medical Oncology, Istituto Scientifico Romagnolo per lo Studio e la Cura dei Tumori (IRST) IRCCS, Meldola, Italy; ^3^ Unit of Biostatistics and Clinical Trials, Istituto Scientifico Romagnolo per lo Studio e la Cura dei Tumori (IRST) IRCCS, Meldola, Italy; ^4^ University of Florence, Florence, Italy; ^5^ The Institute of Cancer Research and The Royal Marsden, London, UK

**Keywords:** enzalutamide, androgen receptor, circulating cell free DNA, copy number variation, prostate cancer

## Abstract

In the present study, we aimed to evaluate the association of circulating *AR* copy number (CN) and outcome in a cohort of patients with advanced castration-resistant prostate cancer (CRPC) treated with enzalutamide after docetaxel. Fifty-nine CRPC patients were evaluated. *AR* CN was analyzed with real-time and digital PCR in the serum collected at starting of treatment. Progressive disease was defined on the basis of Prostate Cancer Working Group 2 criteria. *AR* CN gain was found in 21 of 59 (36%) patients. Median baseline PSA, alkaline phosphatase and lactate dehydrogenase levels were higher in the *AR* CN gained group (*p* = 0.007, *p* = 0.003, *p* = 0.0009, respectively). Median PFS of patients with *AR* CN gain was 2.4 (95%CI: 1.9−3.2) vs. 4.0 months (95%CI: 3.0−6.5) of those with no gain (*p* = 0.0004). Median OS of patients with *AR* CN gain was 6.1 (95%CI: 3.4−8.6) vs. 14.1 months (95%CI: 8.2−20.5) of those with no gain (*p* = 0.0003). At multivariate analysis, PSA decline ≥ 50% and *AR* CN showed a significant association with PFS (*p* = 0.008 and *p* = 0.002, respectively) and OS (*p* = 0.009 and *p* = 0.001, respectively). These findings indicate that the detection of circulating *AR* CN gain is a promising non-invasive biomarker for outcome prediction to enzalutamide treatment in CRPC patients.

## INTRODUCTION

Androgen steroids play a key role in prostate cancer growth and development making the androgen-deprivation the principal therapeutic approach in the different stages of disease. However, hormone therapies lead to a transitory decrease in testosterone and dihydrotestosterone synthesis. Prostate cancer, especially in advanced disease, can only benefit temporarily from these treatments progressing to a castration-resistant prostate cancer (CRPC) status [1]. Despite this resistance to hormonal drugs, androgen signaling remains persistent, therefore targeting the androgen receptor pathway still continues to be a good therapeutic challenge for these patients [2]. Enzalutamide is a next generation anti-androgen with a great affinity for androgen receptor (AR) [3]. The results of two randomized phase 3 trials on enzalutamide *versus* placebo in patients with CRPC before docetaxel treatment (Prevail study) and after docetaxel (Affirm trial), respectively, showed its efficacy in increasing progression-free survival (PFS) and overall survival (OS) [4, 5]. More recently, in two randomized phase 2 studies (Terrain study and Strive trial) enzalutamide significantly reduced risk of progression or death compared with bicalutamide in patients with asymptomatic or mildly symptomatic CRPC [6, 7]. These promising results lead to the increasing use of enzalutamide. Therefore it becomes essential to better understand the mechanisms of drug resistance to select patients who will really experience a treatment benefit in terms of PFS and OS.

AR axis is one of the most important actors in acquiring resistance to androgen targeting therapies. It has been shown that studying specific AR-related alterations could represent an optimal chance for finding predictive markers [8]. Circulating cell free DNA (cfDNA) is a promising, non-invasive source of molecular biomarkers and constitutes a “liquid biopsy” providing a genomic landscape of the cancer at the time of sample taking [9]. For this reason, many efforts have been made for studying aberrations, such as copy number alterations or specific mutations of *AR* in cfDNA, also thanks to the advancement in next generation sequencing technologies [10, 11]. In recent studies, a significant association has been reported between *AR* copy number (CN) in cfDNA and the clinical outcome of metastatic CRPC patients treated with abiraterone [8, 12]. We here aimed to test the association between *AR* CN in cfDNA and resistance to enzalutamide in a cohort of 59 CRPC patients treated with enzalutamide after docetaxel.

## RESULTS

### *AR* copy number gain in CRPC

Fifty-nine patients with metastatic CRPC were treated with enzalutamide between August 2012 and November 2015 and serum samples were collected at starting of treatment. Serum DNA was extracted and was tested for *AR* CN. Median DNA concentration was 4.5 ng/μl (range: 2–23.7 ng/μl). *AR* gene was gained in 21 (36%) patients. Approach sensitivity was previously tested [12]. Clinical characteristics are reported in Table 1. Higher median baseline PSA, alkaline phosphatase and lactate dehydrogenase levels were significantly associated with *AR* CN gain (*p* = 0.007, *p* = 0.003, *p* = 0.0009, respectively). Nine out of 28 (32%) patients previously treated with abiraterone had *AR* gain. This frequency of *AR* gain was similar to the prevalence in patients who had not received abiraterone (39%) (*p* = 0.602), therefore the probability to find *AR* gain in patients with or without previous abiraterone treatment was the same (Table 1). The frequency of *AR* gain was similar in patients with response duration to androgen deprivation therapy less than 18 months (52%) *vs.* those with a response longer than 18 months (38%, *p* = n.s.).

**Table 1:** Patient characteristics

Characteristics	No. cases (%)	AR		*p*-value
		N	A	
		No (%)	No (%)	
Total	59 (100)	38 (100)	21 (100)	-
Median age, y [range]	75 [43–91]	75 [43–91]	74 [66–87]	0.715
Gleason Score				
6–7	20 (34)	14 (37)	6 (28)	0.806
8–9	30 (51)	20 (53)	10 (48)	
Unknown	9 (15)	4 (10)	5 (24)	
ECOG PS				
0–1	56 (95)	36 (95)	20 (95)	0.934
2	3 (5)	2 (5)	1 (5)	
Visceral metastases				
No	51 (86)	32 (84)	19 (90)	0.504
Yes	8 (14)	6 (16)	2 (10)	
No. of previous chemotherapeutic lines				
1	21 (36)	15 (39)	6 (29)	0.406
2 or more	38 (64)	23 (61)	15 (71)	
Previous abiraterone treatment				
No	31 (52)	19 (50)	12 (57)	0.602
Yes	28 (48)	19 (50)	9 (43)	
Median baseline PSA level, ng/mL [range]	68.2 [0.6–4351]	40.5 [0.6–4351]	182.0 [19.8–1443]	0.007
Median baseline ALP level, mU/mL [range]	125 [32–6000]	100 [32–313]	416 [73–6000]	0.003
Median baseline LDH level, mU/mL [range]	204 [122–1808]	194 [122–459]	272 [144–1808]	0.0009

AR, androgen receptor; N, normal; A, amplified; ECOG, Eastern Cooperative Oncology Group; PS, performance status; PSA, prostate-specific antigen, ALP, alkaline phosphatase; LDH, lactate dehydrogenase.

### Association with PSA decline and clinical outcome

A PSA decline ≥ 50% was reported in 18 of the 59 (31%) patients, 4 of 21 (19%) with *AR* gene gain and 14 of 38 (37%) without *AR* gene gain (*p* = 0.260). A PSA decline ≥ 90% was reported in 5 of the 59 (8%) patients, all cases without *AR* gene gain.

**Figure 1:**
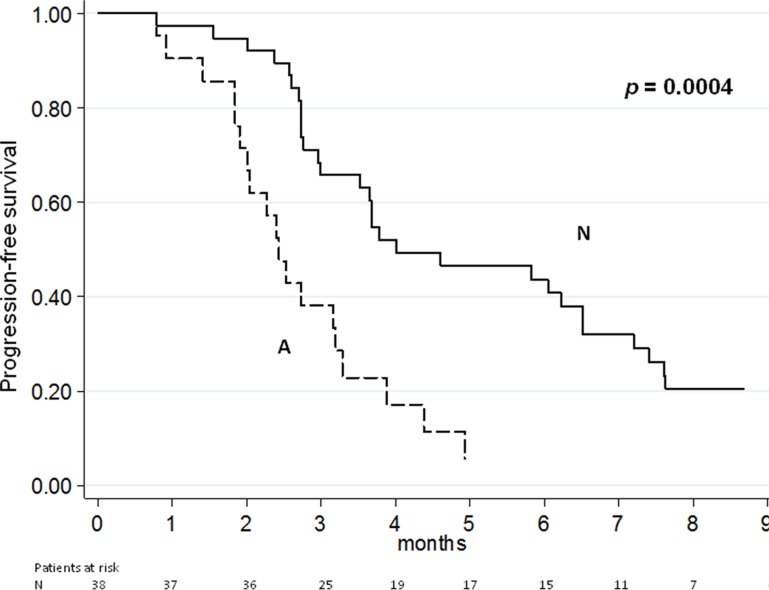
Progression free-survival according to *AR* CN

**Figure 2:**
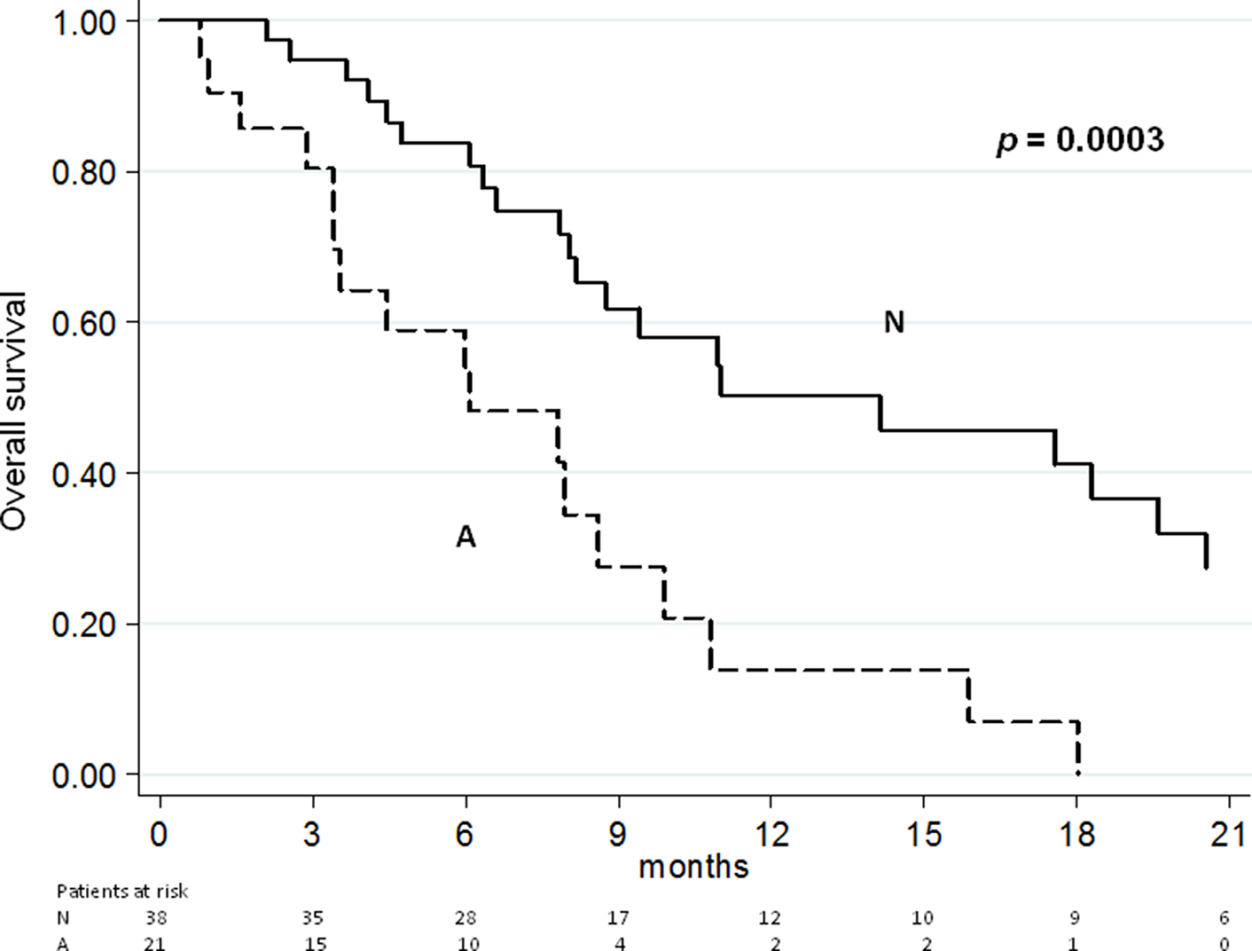
Overall survival according to *AR* CN

At the time of the analysis, 54 of the 59 patients had progressive disease (PD) and 39 patients had died. Median PFS and OS for the overall population were 3.5 months (95% CI: 2.7–4.4) and 9.4 months (95% CI: 7.8–14.1), respectively. The median PFS of patients with *AR* gene gain was 2.4 months (95% CI: 1.9–3.2) *vs*. 4.0 months (95% CI: 3.0–6.5) of those with no CN variation (*p* = 0.0004) (Figure 1). The median OS of patients with *AR* gene gain was 6.1 months (95% CI: 3.4-8.6) compared to 14.1 months (95% CI: 8.2–20.5) for individuals with one copy of the gene (*p* = 0.0003) (Figure 2). Supplementary Figures S1 and S2 show the PFS and OS curves, respectively, made for *AR* gain *vs.* non-gain in patients without abiraterone and *AR* gain *vs.* non-gain with previous abiraterone.

Raw hazard ratios for PFS and OS are summarized in Table 2. Univariate analysis showed a significant association for PFS and OS with patients previously treated or not with abiraterone, PSA decline and *AR* CN (Table 2). At multivariate analysis, the only factors which maintained the association with PFS and OS were PSA decline ≥ 50% (*p* = 0.008 and *p* = 0.009, respectively) and *AR* CN (*p* = 0.002 and *p* = 0.001, respectively). We also analyzed the sensitivity and specificity of *AR* CN in PFS and OS (Supplementary Table S1).

**Table 2:** Univariate analysis for progression-free survival and for overall survival

		Progression-free survival		Overall survival	
		Raw HR (95%CI)	*p*	Raw HR (95%CI)	*p*
**Visceral metastases**	yes *vs*. no	1.57 (0.72−3.41)	0.257	2.44 (1.11−5.37)	**0.027**
**No. of previous chemo lines**	≥ 2 *vs*. 1	1.67 (0.94−2.99)	0.081	2.24 (1.09−4.63)	**0.029**
**Previous abiraterone**	yes *vs*. no	1.97 (1.13−3.46)	**0.017**	2.41 (1.21−4.78)	**0.012**
**PSA decline ≥ 50%**	no *vs*. yes	3.23 (1.71−6.10)	**0.0003**	3.52 (1.59−7.79)	**0.002**
***AR* CN variation**	A *vs*. N	2.79 (1.55−5.02)	**0.0006**	3.23 (1.64−6.35)	**0.0007**

PSA, prostate-specific antigen; AR, androgen receptor; CN, copy number; A, amplified; N, normal.

## DISCUSSION

Analysis of cfDNA is a promising and minimally invasive approach for characterizing the tumor genome in CRPC patients [13, 14]. In this paper, we performed *AR* copy number analysis on cfDNA in CRPC patients treated with enzalutamide after docetaxel. The distribution of median baseline PSA, alkaline phosphatase and lactate dehydrogenase levels were significantly higher in patients with *AR* CN gain, even if a cause-effect relationship cannot be established. Detection of *AR* gain in pretreatment cfDNA was significantly associated with treatment resistance and with shorter PFS and OS.

This study highlighted *AR* gain as an independent biomarker from univariate and multivariate analyses for PFS and for OS. Moreover the multivariate analysis demonstrated PSA decline ≥ 50% could be a predictor of higher PFS and OS. The lack of association between *AR* CN gene and PSA response rate could have an explanation in possible changes of tumor biology during enzalutamide that were not present before treatment (e.g. neuroendocrine differentiation), even if a bias due to the small sample size needs to be considered [15, 16].

Currently, the sequential treatment is a hot topic of CRPC because of increasingly availability of survival-prolonging agents arising the importance of patient selection to improve CRPC management. We here selected a cohort of patients who had all received docetaxel and, in 48% of cases, also abiraterone. As expected, we therefore observed a shorter PFS and OS and lower response rate than reported in the chemotherapy-naïve setting. Our study supported the use of *AR* gain to select patients for enzalutamide but requires repeating in cohorts of patients with different clinical history to confirm that the association with outcome is maintained. Interestingly, prior use of abiraterone was no associated with a higher prevalence of *AR* gain, in keeping with our previous study that reported no significant change in AR status in sequential samples to progression [8].

To allow analysis of all our patients treated in early access program, we used serum samples. Due to concerns with increased leukocyte DNA release, we have more recently collected plasma for cfDNA studies. Increased levels of normal DNA could reduce our ability to detect *AR* CN gain clones and thus, reduce the difference we observed between *AR* gain and normal cancers. Overall, our observation may therefore be shown to be even more significant in future studies.

The only univariate analysis for PFS and OS showed a significant inverse association with prior abiraterone treatment. This confirms the limited antitumor activity of enzalutamide following abiraterone in metastatic CRPC patients irrespective of prior docetaxel use [17]. However, abiraterone before enzalutamide treatment and the number of previous chemotherapeutic lines did not influence the frequency of *AR* CN variation at baseline. These evidences confirmed the lack of changes in AR status during hormonal treatment identified in circulating tumor DNA from 44 CRPC patients treated with abiraterone [8].

In addition, among clinical features of prostate cancer, the presence of visceral metastases has been evaluated in this paper: no correlation with *AR* CN frequency was observed. Conversely, the univariate analysis revealed that the presence of visceral metastases had a significant negative effect only on OS as well as we have shown in our previous work of 256 CRPC patients treated with abiraterone after docetaxel [18].

The small sample size, the retrospective nature of the analysis and the absence of an independent validation set represented the main limitations of our study. Consequently, our results should be considered preliminary and hypothesis-generating. It can be hypothesized that enzalutamide-resistant patients with *AR* CN gain may benefit in terms of survival from chemotherapy and from other therapies that do not target AR signaling. Previously, several studies have already demonstrated that *AR* CN gain together with somatic point mutations were correlated with worse clinical outcome in CRPC patients [8, 10, 12, 19].

Identifying clinical and molecular factors predictive of response to enzalutamide remains a high priority for future research. Recently, Antonarakys *et al*. [20] have demonstrated that a splice variant of *AR*, ARV7, found in circulating tumor cells, was strongly associated with a poor outcome in patients treated with abiraterone or enzalutamide. We believe that also the evaluation of *AR* CN variation could have a similar important impact in guiding clinical decision in CRPC patients.

In conclusion, *AR* CN variation seems to be a biomarker potentially valid from an analytical point of view and pave the way for a further clinical validation in prospective studies on larger number of patients.

## MATERIALS AND METHODS

### Patient cohort

Patients with metastatic CRPC without neuroendocrine differentiation in the primary tumor after at least one chemotherapeutic treatment including docetaxel and treated with enzalutamide were included in this retrospective evaluation in a cohort of patients collected prospectively to study. Additional selection criteria included Eastern Cooperative Oncology Group (ECOG) performance status ≤ 2; adequate cardiac, hepatic, renal and bone marrow function; serum potassium level ≥ 3.5 mmol/L; and ongoing androgen deprivation therapy with serum testosterone < 50 ng/dL. The protocol was approved by the Institutional Review Board. Written informed consent was obtained from all patients.

Treatment consisted of enzalutamide 160 mg daily, that was given continuously until evidence of PD or unacceptable toxicity. Baseline serum PSA levels were typically measured in the week before starting enzalutamide treatment. All recorded PSA test and scan results were collected for these patients: PSA was usually evaluated every 4 weeks for serologic response and imaging investigations as clinically indicated. The Prostate Cancer Working Group 2 (PCWG2) criteria were used to define response and progression [21]. However, in clinical practice deterioration, in clinical conditions and/or radiologic progression according to local radiologist evaluation were also criteria sufficient to establish PD and discontinuation of enzalutamide treatment.

### CN analysis

Serum DNA was extracted with QIAamp DNA Mini Kit (Qiagen, Milan, Italy). DNA was quantified by spectrophotometric evaluation (NanoDrop^®^ ND-1000, Celbio, Milan, Italy). CN analyses were performed by a duplex TaqMan real-time PCR assay (Applied Biosystems, Foster City, CA, USA) for *AR* gene and 2 different reference genes: *RNaseP* (TaqMan Copy Number Reference Assay) and *AGO1* (ID: Hs02320401). Samples analysis was performed as previously described [12]. For *AR*, the cutoff values were > 1.5 for amplification. Data were also analyzed by Digital PCR QuantStudio^®^ 3D System. We performed an absolute quantification of target and reference gene for each sample.

### Statistical analysis

PFS was defined as the time between the first day of enzalutamide therapy and the date of PD or death. Patients without PD at database closure were censored at the final follow up. OS was defined as the time between the first day of enzalutamide and the date of death from any cause or censored at the date of the last follow-up visit.

The association between CN gain and clinical outcome was evaluated by the Kaplan-Meier method and log-rank test. A Cox regression model was used to estimate hazard ratios (HR) and 95% confidence intervals (CI) for PFS and OS. The multivariable Cox models included all factors that were significantly associated in the univariate models. All *P*-values were two-sided and a *P* < 0.05 was considered statistically significant. Statistical analyses were performed with SAS 9.3 software (SAS Institute, Cary, NC).

## SUPPLEMENTARY MATERIALS FIGURES AND TABLE


