# Supplementary material for: Circulating AR copy number and outcome to enzalutamide in docetaxel-treated metastatic castration-resistant prostate cancer
Source: Oncotarget. 2016 May 13;7(25):37839–45. doi: 10.18632/oncotarget.9341 (PMC5122353; doi:10.18632/oncotarget.9341)
Supplement: Supplementary file 1 [file oncotarget-07-37839-s001.pdf]

Circulating *AR* copy number and outcome to enzalutamide in docetaxel-treated metastatic castration-resistant prostate cancer

Supplementary Materials

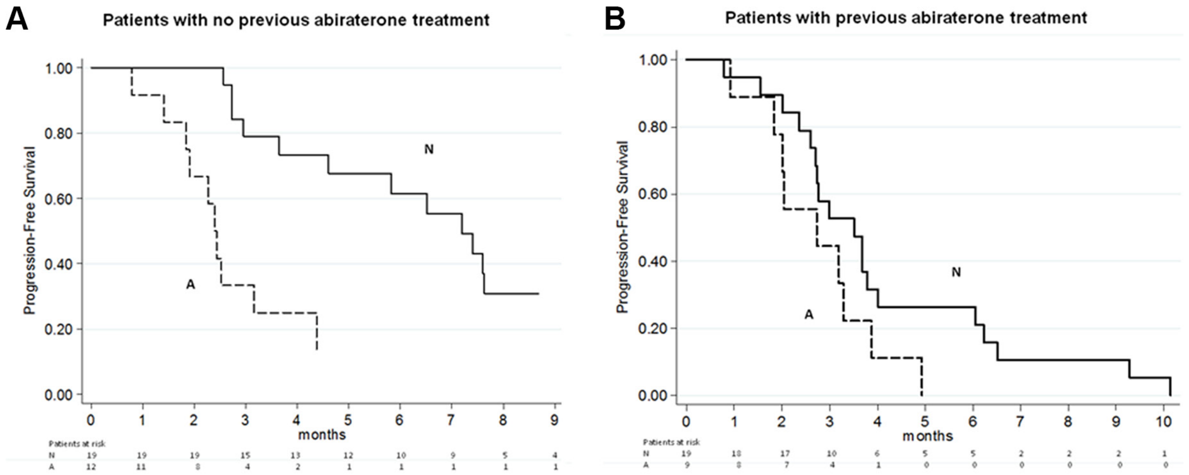

AR CN	patients	Median months PFS (95% CI)	*p*-value
N	19	7.2 (3.7-10.1)	0.0005
A	12	2.4 (1.4-4.4)	

AR CN	patients	Median months PFS (95% CI)	*p*-value
N	19	3.5 (2.6-4.0)	0.117
A	9	2.7 (0.9-3.9)	

Supplementary Figure S1: Progression free-survival curves made for *AR* CN gain *vs*. non-gain in patients without abiraterone and *AR* CN gain *vs*. non-gain with previous abiraterone.

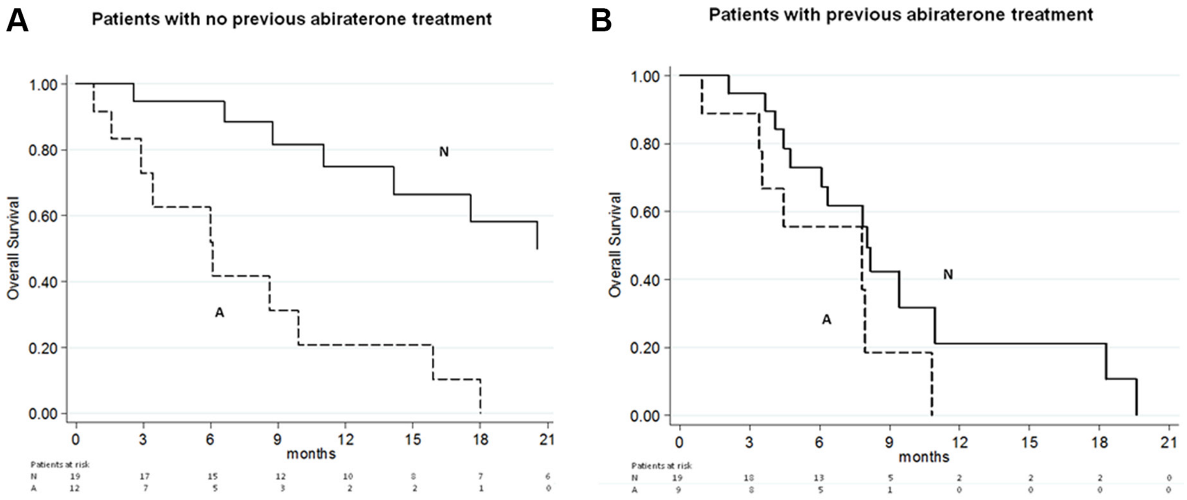

AR CN	patients	Median months OS (95% CI)	*p*-value
N	19	20.5 (11.0-not reached)	0.0002
A	12	6.1 (1.6-9.9)	

AR CN	patients	Median months OS (95% CI)	*p*-value
N	19	8.0 (4.7-11.0)	0.114
A	9	7.8 (1.0-10.8)	

Supplementary Figure S2: Overall survival curves made for *AR* CN gain *vs*. non-gain in patients without abiraterone and *AR* CN gain *vs*. non-gain with previous abiraterone.

Supplementary Table S1: Sensitivity and specificity of *AR* copy number variations with respect to PFS and OS

	Sensitivity (95% CI)	Specificity (95% CI)	Overall Accuracy (95% CI)
**Progression-free survival**			
AR CN	55.2 (41.5−68.1)	85.7 (73.4−93.1)	70.2 (58.2−82.1)
**Overall survival**			
AR CN	50.0 (35.1−64.9)	80.0 (65.1−89.8)	63.0 (49.1−77.0)

AR, androgen receptor; CN, copy number.
